# Progress in Nervous Diseases

**Published:** 1894-06-09

**Authors:** 


### Medical Progress.

### PROGRESS IN NERVOUS DISEASES.

**Syphilitic Spinal Paralysis.**—Erb originally described the symptom complex of this condition as follows: The gait markedly spastic, but the muscular spasm comparatively slight; the tendon reflexes greatly exaggerated; the muscular weakness not proportionate to the disturbance of the walk; there exist almost constantly bladder-weakness, impotence, and sensory disturbances, the cranial nerves as well as the upper extremities escaping; the condition develops in the course of three or four months; the difficulty in walking progresses to a high degree of spastic paresis, rarely to a true paraplegia, which, when it is developed, returns again to spastic paresis; this last point serving, in Erb's opinion, to distinguish these cases from simple transverse dorsal myelitis. Oppenheim[1] considers the clinical picture described by Erb is not indicative of syphilis specially, but that every other form of diffuse lesion of the dorsal cord can produce the same clinical appearance. Oppenheim considers the chief form of spinal syphilis is a general meningo-myelitis; he relies for differential diagnosis on the following points: (1) Previously or simultaneously existing brain symptoms; (2) the interrupted course of the disease and the fluctuation of the individual symptoms; (3) symptoms of meningeal irritation and of involvement of the roots; (4) phenomena indicating multiplicity of foci; (5) the incomplete development of "Brown-Sequard's paralysis." Gerhardt[2] thinks that of the structures within the spinal canal the meninges and the blood vessels of the cord are most apt to be the seat of syphilitic disease, though gummata are not extremely rare, and when laterally placed give rise to "Brown-Sequard's paralysis." The meningitic processes in the cord are associated often with a similar affection of the cerebral meninges, in which condition he has twice observed "triplegia," *i.e.*, paraplegia and hemiplegia combined, but most of the cases resemble more closely locomotor ataxia. He has seen four cases of Erb syphilitic spinal paralysis, as above described, but does not think that as yet they are sufficiently explained anatomically. Occlusion of the arteries from specific disease of the coats will lead in these cases to a sudden or "apoplectiform" increase of the spinal paralysis. As additional forms of spinal syphilis Gerhardt mentions poliomyelitis syphilitica, cases of Landry's paralysis and multiple affection of the spinal nerve-roots. Besides the primary affection the nerve-roots may be involved in a spinal meningitis, in which manner unusual localised lesions are produced at times. The interval of time between primary infection and the onset of spinal symptoms varies from three months to ten or twenty years. Early diagnosis and persevering anti-syphilitic treatment gives fairly successful results; in one of the cases of locomotor ataxy treatment restored the tendon reflex and the light reflex of the iris. H. Lamy[3], discussing syphilitic meningo-myelitis, agrees substantially with Gerhardt. His conclusions are, first, anatomically the common type observed is a diffuse meningo-myelitis with changes in the vessels of the cord, either lepto-meningitis simply or with a pachymengitis also, the latter nearly always being present in the cervical region; similar changes are found in the brain, especially at the base in the region of the interpeduncular space and chiasma. The commencement of the vascular lesion is in the tunica adventitia, the veins being more affected than the arteries. Second, clinically the best marked forms present two stages of development, corresponding to localisation of the lesion in the membranes alone first and in the cord also afterwards. The prodromal period is characterised by sensory affections in connection with meningitis, and consist chiefly of rachialgia. At this period cerebral complications are frequently present. The second period is characterised by spinal paralyses, which most commonly present the clinical aspect of a transverse dorsal myelitis of medium severity—incomplete spastic paralysis with affection of the sphincters. The syphilitic nature of the malady may be suspected by the character of the paralysis itself, by marked peculiarities of the prodromal period and by the presence, or history, of cerebral lesions. Occasionally paralyses develop with great rapidity. After a short prodromal period acute central myelitis develops, terminating fatally in a month or less. The specific lesions found on anatomical examination leave no doubt as to the nature o the morbid process.

**Epilepsy.**—The relationship between epilepsy on the one hand and errors of refraction and of the action of the ocular muscles on the other hand continues to be discussed without substantial agreement between different authorities being arrived at. H. W. Dodd[4] reports upon one hundred cases of epilepsy, their refraction, and their treatment by glasses. He finds that the epileptic seem to be less frequently hypermetropic, but more frequently astigmatic than those who are not epileptic, and draws the conclusion that, given a certain condition of instability of the nervous system, (*a*) errors of refraction may excite epilepsy; (*b*) the correction of errors of refraction will in nearly half the cases cure or relieve the epileptic condition. (*c*) Epilepsy will continue generally in a modified form when the error of refraction has been corrected, even though that error has been the original exciting cause. Ranney[5], insisting on the necessity of the correction of eye-defects in epileptics, remarks that to anyone familiar with the later methods of investigation those of Mr. Dodd must seem singularly incomplete, and that the principle of treatment of convulsive seizures by drugs is wrong. He lays more stress upon errors in the action of the ocular muscles (heterophoria) than upon errors of refraction, as being the cause of attacks in the epileptic. He considers that (*a*) in epilepsy examination of the eyes (for errors of refraction) and of the eye-muscles (for heterophoria) is the most important step in the search for sources of reflex disturbance. (*b*) Investigation must be made by one thoroughly familiar with the later methods for the determination of "latent" heterophoria. (*c*) It is safe to hope that a radical correction of marked heterophoria and abnormal refraction will eventually be followed by decided benefit, the degree of benefit being greater than that obtained by the exhibition of bromides. (*d*) Other sources of peripheral irritation must be sought for when no relief has been obtained by eye-treatment. (*e*) The duration of treatment of the heterophoria by graduated tenotomy may last two or three years. (*f*) The danger of over-correction in operation for heterophoria is very small, and when produced is easily controlled and rectified. G. T. Stevens[6], in a paper read before the Pan-American Medical Congress, strenuously supported the same views as those advanced by Ranney, and he dwells still more upon the protracted length of time over which it is often necessary to continue the treatment by graduated tenotomy:—he is "sometimes asked by his colleagues if there is no end to the treatment. Does not the condition, which has been apparently removed, return?" These views, however, do not meet with general acceptance even in New York. R. K. Macalister[7] reporting upon 250 cases of epilepsy observed in Professor Dana's clinic, enumerates the predisposing and exciting causes which were found to exist in these cases; no mention is made either of errors of refraction or of abnormal action of the eye-muscles as causing epileptic attacks, and as regards treatment the opinion given is that "our old stand-by the bromide treatment still ranks first in our therapeutic armamentarium." The question of the effect of correcting errors of refraction in epileptics was brought before the British Medical Association at their last annual meeting. Dr. John Heron[8] there reported three cases successful out of five in whom the errors of refraction had been corrected by lenses. Others present had met with less success. Mr. Cant had obtained some relief in two cases out of many, and Dr. Percival "had no successes to report" out of a fair number of cases sent to him. While, therefore, it is obvious on the one hand that as yet no general concord has been arrived at respecting the influence of eye-strain on epilepsy, on the other it is more truly scientific to endeavour to remove the exciting cause of the attacks than simply to benumb the reflex irritability of the nerve-centres by administering bromides indiscriminately to all cases of the disease.

**Cerebellum.**—Hughlings-Jackson, and Risien Russell,[9] in commenting upon an interesting case in which post-mortem there was found a cystic condition of the cerebellum (both lateral lobes being involved), maintain that the disorders of locomotion seen in cerebellar disease depend not upon an ataxy, but upon a true paresis of the trunk muscles, these being first and most affected, then the muscles of the inferior extremities, and those of the superior extremities last and least. In this case the paretic condition of the back muscles was shown by the development of "saddle-back" (lordosis) on standing with the feet close together, and by the defective power of raising the head from the ground when lying flat in the prone position; the weakness of the abdominal muscles was shown similarly by the inability to rise from the supine position without the aid of the hands and arms. There was no appreciable paresis of the limbs. H. Jackson thinks that the cerebellar reel is due to the erratic movement of the legs attempting to "prop up" the trunk in its immoderate inclinings, resulting from weakness of the spinal muscles. It has been said that no motor connections between the cerebellum and spinal cord have been discovered. Marchi has recently described descending degeneration after lesion of the cerebellum in dogs and monkeys. Risien Russell has found in some cases (but not in others), after ablation of one lateral half of the cerebellum, degenerated fibres in the cord mainly in the antero-lateral region on the same side as the lesion. Ferrier and Aldren Turner,[10] however, have been unable to confirm Marchi's statement as to the existence of a direct efferent cerebellar tract in the spinal cord, or of degeneration of the anterior nerve roots after extirpation of the cerebellum in the monkey. (Death in this case of Jackson's and Russell's was from apnœa, the heart continuing to beat eight and a-half hours after the cessation of natural respiration. The state of spinal cord does not appear to be recorded.) A. W. Campbell[11] records the condition of the nerve centres of a case where death took place one year after the occurrence of thrombosis of the left inferior cerebellar artery. In the cord, after hardening and staining, degenerated fibres were found in the sacral cord in the crossed pyramidal tract of the left side; at higher levels these patches increased in size, the direct cerebellar tract being extensively diseased, and the crossed pyramidal tract slightly so. In the upper dorsal region the antero-lateral tract was also affected, and the cells in Clarke's column diminished in numbers. In the cervical region a trace of degeneration was found in the opposite antero-lateral tract; degenerated fibres were also found in both the anterior and posterior nerve-roots along the cord. Campbell thinks this case proves the existence of downward conducting paths from the cerebellum in the cord, but the value of the case is detracted from by the fact that the internal capsules and other parts above the cerebellum do not appear to have been examined microscopically. Royet and Collet[12] record a case of sclerosis of the cerebellum, in which the symptoms during life had been identical with those of multiple cerebro-spinal sclerosis, and there had been also tremor of the vocal cords. Nothing abnormal, however, was found in the spinal cord or the hypoglossal root-fibres and nuclei.

**Hypnotics.**—*Chloralose* has been recently the subject of recorded experience not wholly favourable. Ohmjelewski[13] thinks it a valuable hypnotic in mental cases in 4-8 grain doses. Excitement and tremor of the upper limbs were sometimes observed to occur. Watson Williams[14] records a case in which maniacal symptoms lasting several hours followed the administration of 10 grains, the pulse, however, remained "unusually good." Dr. G. Bardet on several occasions has contributed information about chloralose to the Therapeutical Society of Paris[15]. His original view was that all the chloral derivations (hypnal, somnal, chloralose, &c.), produced simply the same effect as chloral, and that 12 grains of chloralose equalled 8 grains of chloral. Subsequently he withdrew this opinion, as he had two cases in which, besides the hypnotic effect, excito-motor phenomena were induced, intense dyspnæa and extreme agitation following a dose of 8 grains. Notwithstanding the statements of Féré[16], Bardet thinks that a dose of 8 grains should never be exceeded, and that the only advantage chloralose has is that it does not depress the heart's action.

*Trional* appears so far to have manifested no untoward properties. Beyer[17] records it as effectual in producing refreshing sleep in doses of 15-25 grains, which quantity may be doubled if acute mania is present. J. B. Mattison[18] considers trional the "best hypnotic" after having given it hundreds of times. It is not an anodyne, and in action is most nearly approached by sulphonal, than which it is more prompt and more prolonged in effect. Mattison finds it specially useful in the early abstinence time in the treatment of narcotic inebriety.

[1] Berl. Klin. Woch., No. 35, 1893. [2] Berl. Klin. Woch., No. 50, 1893. [3] Nouviconograph de la Salpê, No. 2, 3, 4, 5, 1893. [4] Brain, Winter, No., 1893. [5] New York Med. Journ., Jan. 13th, 20th, and 27th, and Feb. 17th, 1894. [6] New York Med. Journ., Nov. 1893. [7] New York Med. Journ., Jan. 27th and Feb. 24, 1894. [8] Brit. Med. Journ., Sept. 30th, 1893. [9] Brit. Med. Journ., Feb. 24th, 1894. [10] Trans. Roy. Soc., 1891. [11] Liverpool Med. Journ., Jan., 1894. [12] Arch-de-Neurol., quoted in B.M.J., Jan. 20th, 1894. [13] Quoted in Ep. B.M.J., March 10th, 1894. [14] Pract., Feb., 1894. [15] Les Nouveaux Remedes, Jan. 24th, and Feb. 24th and Med. Week, Feb. 23rd, 1894. [16] Soc. de Biologie, March, 1893. [17] Quoted in Clin. Journ., Feb. 7th, 1894. [18] New York Med. Journ., Jan. 13th, 1894.

